# The Behavior Change Techniques Used in Canadian Online Smoking Cessation Programs: Content Analysis

**DOI:** 10.2196/35234

**Published:** 2022-03-01

**Authors:** Laura Struik, Danielle Rodberg, Ramona H Sharma

**Affiliations:** 1 School of Nursing University of British Columbia Kelowna, BC Canada; 2 School of Social Work University of British Columbia Kelowna, BC Canada

**Keywords:** content analysis, smoking cessation, internet, behavior change technique, mental health, smoking, online program, website, government, federal, provincial

## Abstract

**Background:**

Smoking rates in Canada remain unacceptably high, and cessation rates have stalled in recent years. Online cessation programs, touted for their ability to reach many different populations anytime, have shown promise in their efficacy. The Government of Canada has therefore funded provincial and national smoking cessation websites countrywide. However, little is known about the behavior change techniques (BCTs) that underpin the content of these websites, which is key to establishing the quality of the websites, as well as a way forward for evaluation.

**Objective:**

The purpose of this study, therefore, is to apply the BCTTv1 taxonomy to Canadian provincial and federal websites, and to determine which BCTs they use.

**Methods:**

A total of 12 government-funded websites across Canada were included for analysis. Using deductive content analysis and through training in applying the BCTTv1 taxonomy, the website content was coded according to the 93 BCTs across the 16 BCT categories.

**Results:**

Of the 16 BCT categories, 14 were present within the websites. The most widely represented BCT categories (used in all 12 websites) included goals and planning, social support, natural consequences, and regulation. Implementation of BCTs within these categories varied across the sites.

**Conclusions:**

Analyzing the content of online smoking cessation websites using the BCTTv1 taxonomy is an appropriate method for identifying the behavior change content of these programs. The findings offer programmers and researchers tangible directions for prioritizing and enhancing provincial and national smoking cessation programs, and an evaluation framework to assess smoking cessation outcomes in relation to the web-based content.

## Introduction

Tobacco use is the leading cause of preventable disease, disability, and death globally. In Canada, 45,000 people die every year due to a tobacco-related illness [1]. Although cigarette smoking has decreased overall in Canada [2], rates of current smoking remain unacceptably high at 15% [3]. In addition, the rise in e-cigarette use has added concerns about nicotine addiction from e-cigarettes that will eventually transfer to smoking [4], and there is some evidence indicating that the increase in e-cigarette use parallels an increase in smoking uptake among younger demographics [5]. Even further, recent evidence indicates that smoking cessation has stalled since the COVID-19 pandemic. According to a cross-sectional research study across Australia, Canada, England, and the United States, although nearly 50% of smokers indicated that they were thinking about quitting due to the pandemic, changes in smoking status were marginal: only 1.1% attempted to quit, 14.2% reduced smoking, 14.6% increased smoking, and 70.2% reported no change [6]. This is due, in part, to reduced access to smoking cessation treatment and support. For example, a study of the Ontario STOP smoking program, which transitioned from in-person clinic visits to online visits during the pandemic, found that enrollment decreased by 69% and that there was a 42% drop in visits in the spring of 2020 compared to the previous 2 years [7].

Given that one life can be saved for every two people who quit smoking [8], making cessation supports available and accessible to smokers is critical. Online smoking cessation programs are known for their low costs per smoker, accessibility, availability, and their potential to reach a large proportion of smokers [9]. Given that 75% of Canadians 15 years and older reported an increase in internet use since the pandemic [10], the value of online-based cessation support becomes foregrounded. Although there is evidence to support the efficacy of online cessation programs, especially interactive ones, researchers repeatedly emphasize the need to ensure the quality of these interventions to avoid disappointment and failed quit attempts [11-13]. For example, a systematic review that aimed to examine the efficacy and effect modifiers of eHealth interventions for smoking cessation found that, after pooling findings from 67 web-based cessation programs, compared to nonactive control conditions (eg, print materials, assessment only, or no intervention), web-based programs were significantly more effective at 6 months [13]. However, the authors also cautioned that the quality of the web-based programs modified the effect of the program (poorer quality programs yielded fewer positive outcomes) [13].

One way to establish and assess the quality of online smoking cessation interventions is by articulating the behavior change techniques (BCTs) that form the basis of a program’s content [14]. The BCT taxonomy provides a list of methods that could be used in a program to yield behavior change [14-16] (Table 1). The BCTTv1 taxonomy consists of 93 BCTs in 16 categories that address the different targets of behavior change, including capability, opportunity, and motivation [17,18]. The benefit of web-based intervention programs is maximized when the BCTs included have been shown to be effective. For example, in England, researchers were able to identify BCTs that were associated with cessation success rates in local smoking cessation services [19]. This subsequently informed guidance on service provision and learning objectives in training courses, which was associated with increased success rates of smokers who were engaged in these services [20]. Therefore, articulating the BCTs used in smoking cessation programming is of utmost importance to understand what works for whom and how.

The Canadian government funds a comprehensive set of online provincial and national smoking cessation programs. However, little is known about how these programs are designed to influence smoking cessation behavior in relation to the BCTs and subsequently how to leverage strengths and address weaknesses. Understanding this is critical, especially in the context of an increasingly complex tobacco use landscape, so that we can reach the national goal of less than 5% tobacco use by 2035 [1]. The purpose of this study, therefore, was to apply the BCTTv1 taxonomy to Canadian provincial and federal websites and determine which BCTs they use.

**Table 1:** Behavior change techniques (BCTs).

BCT category (n=16)	BCTs (n=93), n	Definition/meaning
1. Goals and planning	9	Develop a goal for a behavior or an outcome of a behavior and determine what factors need to be assessed to work toward that goal. This can include periodically reviewing the goal and making verbal/written commitments to work toward the goal.
2. Feedback and monitoring	7	Monitor the progress made with the behavior or outcome of a behavior either by the individual themselves, by others, or by a device. When monitored by others, feedback may or may not be given to the individual being monitored.
3. Social support	3	This can include social support for three reasons: practical purposes like getting to an appointment, emotional purposes like comforting an individual at an appointment, and unspecified purposes like encouraging an individual to attend their appointment.
4. Shaping knowledge	4	Clarify proper performance of the wanted behavior and determine antecedents associated with the unwanted behavior and causes of the unwanted behavior.
5. Natural consequences	6	Provide information on the consequences associated with the unwanted behavior including health consequences, social and environmental consequences, and emotional consequences, which may also include the individual monitoring their emotional consequences.
6. Comparison of behavior	3	Demonstrate proper performance of the wanted behavior and showcase the performance and opinions of others on the wanted behavior.
7. Associations	8	Increase facilitators for the wanted behavior such as prompts/cues and reduce interest in the unwanted behavior and decrease barriers to the wanted behavior such as nagging and fear.
8. Repetition and substitution	7	Practice performing the wanted behavior to develop a new habit to replace the unwanted behavior. This includes gradually increasing the amount the wanted behavior is performed until it becomes a habit.
9. Comparison of outcomes	3	Identify the pros and cons of continuing the unwanted behavior, including identifying future outcomes that will result from the unwanted behavior. Obtaining information from credible sources like health professionals can help identify this.
10. Reward and threat	11	Reward individuals or give them the incentive that they will be rewarded either when the goal is completed or when effort has been put in toward reaching the goal. This can also include individuals rewarding themselves.
11. Regulation	4	Certain resources can be used to aid in reaching the goal by assisting with maintaining a positive mindset such as medications and stress management techniques.
12. Antecedents	6	Modify the social and physical environment to make it conducive for the wanted behavior and unconducive for the unwanted behavior.
13. Identity	5	Change one’s self-identity and beliefs to associate with the wanted behavior rather than the unwanted behavior.
14. Scheduled consequences	10	Eliminate rewards if unwanted behavior occurs and only provide rewards for the wanted behavior in specific circumstances.
15. Self-belief	4	Build the confidence needed to perform the wanted behavior through positive self-talk and persuasion from others as well as focusing on one’s past success and envisioning future success.
16. Covert learning	3	Envision the future consequences of the unwanted behavior and the future rewards of the wanted behavior as well as focus on the consequences and rewards others are currently receiving.

## Methods

### Data Collection

Government-funded websites were found using Google search phrases, such as “smoking cessation Canada provinces and territories” and “smoking cessation federal Canada.” We also searched each individual province with the phrase “smoking cessation.” The exclusion criteria included websites that only had telephone numbers, websites intended for use outside of Canada, websites with information but not interventions (eg, fact sheets), and websites with only government legislation pages (eg, tobacco control acts). The first 10 pages of the search were scanned for provincial and federal smoking cessation websites. Provincial and federal government health websites were entered to find the hyperlinks for tailored websites on provincial or national smoking cessation. Provinces and territories that did not show up in the Google scan were individually searched. The search yielded 6 provinces and 3 territories with tailored websites, in addition to 1 website designed to provide cessation information for a combination of 4 provinces and 1 territory. Furthermore, 2 federal websites were identified, resulting in a total of 12 websites.

### Data Analysis

We used deductive content analysis to determine which BCTs were used in the 12 programs. Deductive content analysis is the process of applying data to a pre-existing framework [21]. All three researchers were familiar with the BCT taxonomy, and one researcher was trained through the BCT online training course, the latter of which did the primary coding of the websites. Through the creation of a document detailing the decision-making processes around applying the BCT taxonomy to each website, a clear audit trail was generated and frequently consulted by the larger team. Points of confusion were discussed, and consensus was reached through consultation with the BCT taxonomy descriptions and the relevant website section.

## Results

### Website Details

The 12 websites represented in this paper include QuitNow (BC), Alberta Quits (AB), Tobacco Free Quebec (QC), New Brunswick Anti-Tobacco Coalition (NB), Tobacco Free Nova Scotia (NS), Smokers Help (NL), Nunavut Quits (NU), Quitpath (YT), North West Territories Quitline (NT), Canadian Cancer Society Smokers’ Helpline (YT, SK, MB, ON, and PE), Quit Smoking (federal), and Break It Off (federal). All 12 websites addressed combustible cigarettes when discussing nicotine cessation. A total of 10 websites included information on vaping, and 6 websites included information on smokeless tobacco, which encompasses both shisha/hookah and chewing tobacco. Only five websites included informational tabs tailored to specific populations. The specific populations addressed ranged from broader categories, such as women, teens/youth, and older adults, to narrower categories, such as First Nations/Inuit/Metis, pregnant and breastfeeding individuals, individuals with mental illness, and individuals with a cancer diagnosis.

All 12 websites had some method of direct contact support for users: 12 websites offered phone support, 6 offered email support, and 5 offered text support. In addition, the 2 federal websites provided links to the 10 provincial and territorial websites for local support. A total of 9 websites had some type of online community. There were a variety of online communities: Facebook (n=8), Twitter (n=6), Instagram (n=4), YouTube (n=2), website forum (n=4), and 1 website offering a video call online support group. The structure of the websites varied following the number of tabs on the home page. The topics of the tabs included the following: quitting (why quit and how to quit), staying quit, community/support, helping others quit, resources, special concerns, contact, and feedback. The number of tabs on each website included 3 tabs (n=4), 4 tabs (n=3), 6 tabs (n=2), 7 tabs (n=1), 2 tabs (n=1), and 5 tabs (n=1).

### BCT Category Representation

The number of BCT categories used in a single website ranged from 5 to 13, with an average of 11 (SD 2.01; see Table 2). All 12 websites include the BCT categories goals and planning, social support, natural consequences, and regulation. This meant that at least one BCT in each of those categories was included in all 12 websites. The least represented categories (in six or fewer websites) included feedback and monitoring, comparison of behavior, and self-belief. The two BCT categories not represented at all include scheduled consequences and covert learning.

**Table 2:** Behavior change technique (BCT) category representation.

BCT category	Websites (n=12)												
	BC	AB	QC	NB	NS	NL	NU	YT	NWT	CCS^a^	QS^b^	BIO^c^	Total, n
													
1. Goals and planning	✓^d^	✓	✓	✓	✓	✓	✓	✓	✓	✓	✓	✓	12
2. Feedback and monitoring			✓		✓	✓	✓		✓			✓	6
3. Social support	✓	✓	✓	✓	✓	✓	✓	✓	✓	✓	✓	✓	12
4. Shaping knowledge	✓	✓	✓		✓	✓	✓	✓	✓	✓	✓	✓	11
5. Natural consequences	✓	✓	✓	✓	✓	✓	✓	✓	✓	✓	✓	✓	12
6. Comparison of behavior	✓	✓					✓	✓	✓				5
7. Associations	✓	✓	✓		✓	✓				✓	✓	✓	8
8. Repetition and substitution	✓	✓	✓		✓	✓	✓	✓	✓	✓	✓	✓	11
9. Comparison of outcomes	✓	✓	✓		✓	✓	✓	✓	✓	✓	✓	✓	11
10. Reward and threat	✓	✓	✓		✓	✓	✓	✓	✓	✓	✓	✓	11
11. Regulation	✓	✓	✓	✓	✓	✓	✓	✓	✓	✓	✓	✓	12
12. Antecedents	✓	✓	✓		✓	✓	✓	✓			✓	✓	10
13. Identity	✓			✓	✓	✓	✓		✓		✓		7
14. Scheduled consequences													0
15. Self-belief		✓			✓	✓		✓		✓	✓		6
16. Covert learning													0

^a^CCS: Canadian Cancer Society.

^b^QS: Quit Smoking.

^c^BIO: Break It Off.

^d^The checkmark indicates that this BCT was used.

### BCT Representation and Implementation

The number of BCTs used in a single website ranged from 8 to 33, with an average of 21.91 (SD 5.85). The number of BCTs represented within each category varied. For example, 5 of the 9 BCTs under goals and planning were used. The ways in which each BCT was implemented also varied within the websites. For example, although all 12 websites used goal-setting (behavior), this BCT was implemented in variable manners (eg, setting a quit date, setting goals for cigarette consumption, or taking a readiness quiz and taking an addiction test to shape a quit plan). The complete representation of the BCTs is listed in Textbox 1 along with a list of ways in which the BCTs were implemented.

**Behavior change technique (BCT) representation and implementation.**

**1. Goals and planning**

1.1 Goal-setting (behavior)

- Setting a quit date
- Providing users with an addiction level test
- Providing users with a confidence in quitting test
- Providing users with a readiness to quit test
- Giving users small goals/reduction exercises

1.2 Problem-solving

- Assistance with identifying triggers/roadblocks
- Relapse prevention strategies (eg, activity)
- Tips on managing cravings and withdrawal

1.3 Goal-setting (outcome)

- Encouraging social media engagement around quit goal

1.4 Action planning

- Making “if/then” plans (eg, if I have a craving, then I will go for a walk)

1.9 Commitment

- Making self-commitment statements (eg, “I will...”)

**2. Feedback and monitoring**

2.3 Self-monitoring of behavior

- Journaling cravings exercise
- Journaling triggers
- Journaling progress to plan self-rewards
- Journaling quit journey

**3. Social support**

3.1 Social support (unspecified)

- Quit Coaches phone and live chat
- Quitline
- Support groups
- Community forum
- Local help directory/map for local support/in-person clinics
- Social media network (Facebook, Instagram, and Twitter)
- Mental health professional helpline
- Advice for friends and family to support
- Email support
- Text messaging support for up to 3 months
- Quit buddy
- Pregnancy support
- Quit connection referral form
- Quit stories

**4. Shaping knowledge**

4.1 Instruction on how to perform the behavior

- Visual instructions on use of nicotine replacement therapy (NRT) products
- Instructions on how to navigate the website
- Instructions on how to navigate second-guessing
- Instructions on how to remove smoke from the home
- Instructions on how to prevent weight gain after a quit
- Instructions for families on how to talk to a smoker
- Instructions for youth and young adults on how to quit
- Instructions on how to use the quitline
- Links to self-help resources/apps

4.2 Information about antecedents

- Tracking antecedents to smoking (eg, nicotine cravings)
- Advice on how to manage a craving

**5. Natural consequences**

5.1 Information about health consequences

- Interactive diagram on the health risks of smoking
- Health risks of nicotine product use
- Risks to pregnancy and breastfeeding
- Risks to cancer recovery
- Links and resources to risks

5.2 Salience of consequences

- Graphic images on the health risks of smoking

5.3 Information about social and environmental consequences

- Second- and thirdhand smoke risks to children and pets
- Economic, environmental, and social effects of smoking
- Increased risk of kids taking up smoking

5.4 Monitoring of emotional consequences

- Recording emotions in a journal while quitting

5.6 Information about emotional consequences

- Negative impacts on quality of life and enjoyment

**6. Comparison of behavior**

6.1 Demonstration of the behavior

- Stories from ex-smokers (eg, videos on other’s quit smoking journey)

**7. Associations**

7.1 Prompts/cues

- Put up a reminder list with the reasons you quit smoking

7.5 Remove aversive stimuli

- Inform friends/family to not nag

7.6 Satiation

- Mindfulness exercises/videos

**8. Repetition and substitution**

8.1 Behavior practice/rehearsal

- Complete a practice quit day

8.2 Behavior substitution

- Suggestions for substitutions to smoking (eg, fruit)

8.7 Graded tasks

- Gradual smoking reduction plan

**9. Comparison of outcomes**

9.1 Credible source

- Quit coaches, doctors, health care providers, pharmacists

9.2 Pros and cons

- Pros and cons list for quitting smoking

**10. Reward and threat**

10.1 Material incentive (behavior)

- Calculator for money spent on cigarettes

10.2 Material reward (behavior)

- Providing milestone certificates

10.3 Nonspecific reward

- Calculating reward of physical improvements

10.6 Nonspecific incentive

- Incentive of benefitting baby/child/family

10.9 Self-reward

- Encouraging self-reward (eg, purchase special gift)

**11. Regulation**

11.1 Pharmacology

- Information on pharmacological support (eg, NRT)
- NRT for pregnancy and youth

11.2 Reduce negative emotions

- Stress management techniques
- Information around management of mental health
- Tips on managing cravings and withdrawal

**12. Antecedents**

12.1 Restructuring the physical environment

- Remove ash trays, remove odor

12.2 Restructuring the social environment

- Hang out with nonsmokers, ask people not to smoke, avoid social situations of temptation

12.3 Avoidance/reducing exposure to behavior cues

- Avoid triggers (eg, change of routine, ensure relaxation)

12.4 Distraction

- Use distractions (eg, keep mind and hands busy)

12.5 Adding objects to the environment

- Prepare by having snacks or gadgets available

**13. Identity**

13.1 Identification of self as role model

- Identifying self as a role model (eg, to kids, to social groups)

**15. Self-belief**

15.1 Verbal persuasion about capability

- Encourage family and friends to let smoker know they believe in them

15.2 Mental rehearsal of successful performance

- Imagining life permanently smoke-free

15.3 Focus on past success

- Focus on past successes when relapsing

15.4 Self-talk

- Encourage smoker to use positive self-talk

## Discussion

### Principal Findings

This is the first study to apply the BCT taxonomy to Canadian government-funded smoking cessation websites. This analysis enables unique comparison and education on a national scale. One major benefit of this analysis is that it provides a framework for understanding which BCTs are used across the nation and how; this provides individual programmers with ideas for strengthening their websites, enables the identification of priority BCTs to include in Canadian cessation programming, and offers a foundation for evaluating strengths and weaknesses in the programs.

Although understanding the overall BCT categories that are being used is helpful and directs programmers to the most widely used BCTs, the granular analysis of BCTs used within each category provides a window into the nuances of how a BCT category and BCT can be executed in an online program. For example, although the BCT information on health consequences under the natural consequences BCT category is used in all 12 websites, its use varied (some provided a text-based list of negative health impacts, while others provided an interactive diagram). This not only cues programmers to the importance of including this BCT in their program but also provides them with ideas for expanding and innovating how to incorporate this BCT in their program (eg, incorporating interactive features).

This analysis also closes a major methodological gap in analyzing provincial and national websites aimed at addressing the same health behavior (smoking). Applying the taxonomy in this context enabled the ability to analyze complicated (ie, nuanced, complex, comprehensive, and tailored) websites, which provides an accessible understanding of the underlying mechanisms that underpin the content to lend to behavior change. In simpler terms, the analysis reveals “how” these websites function.

Further, this analysis provides an evaluation framework for smoking cessation websites. Few websites are evaluated for their effect on behavior change, possibly because websites are more comprehensive and nuanced compared to a single intervention. The first step to investigating the association between the content of the websites and smoking outcomes is the need for a reliable method to describe the content [22]. In this vein, the findings of this study provide researchers with an evaluation framework to investigate the effects of these initiatives. For example, by mapping out the strategies that an individual website uses with regard to the BCT taxonomy and comparing that to the national compilation provided here, researchers could develop survey or interview questions surrounding user experiences with those strategies and if they lent to smoking reduction/cessation. In sum, the findings provide researchers with an evaluation framework that can be used to explore strengths and weaknesses of each technique used within a website with end users, informing what techniques work for whom and how.

Attention to the most widely used (represented in all 12 websites) BCT categories and their associated BCTs is warranted. The most widely used included goals and planning, social support, natural consequences, and regulation. Previous research evidence supports use of most of these BCTs to lend to higher cessation rates. In one study that examined BCT categories used in online interventions and mobile-based interventions and their association with cessation rates, the authors found that five BCT categories were linked to higher cessation rates [23]. These included goals and planning (eg, advice on coping), reward and threat (eg, self-rewards), regulation (eg, advice on pharmacotherapy), antecedents (eg, advice on changing routines), and identity (eg, supporting identity change of being an ex-smoker) [23]. In a study that included 6 online smoking cessation websites evaluated for their effectiveness, cost-effectiveness, and theoretical underpinnings (use of BCTs), the effects indicated that smokers using an online cessation intervention are 1.15 to 2.84 times more likely to become a former smoker compared to the control condition. The majority of the interventions used most, if not all, of these five BCT categories [11]. Finally, in a recent systematic review and meta-regression analysis of BCT categories and BCTs associated with smoking cessation in smoking cessation trials, the authors found 29 individual BCTs as potentially important predictors of smoking cessation in at least one analysis (controlling for total BCTs in one) [24]. Of these, three consistently predicted higher smoking cessation rates and included goals and planning (prompting commitment), reward and threat (social reward), and identity (identity associated with changed behavior) [24]. These results, in combination with this study’s findings, add to a growing body of evidence that supports focusing, expanding, and innovating the use of these BCT categories and their associated BCTs within provincial and national websites.

The findings also highlight areas for improvement and ideas for how improvements could be made by listing the full range of ways in which the BCTs were implemented across the websites. According to the findings and in comparison with previous research findings, there is room to grow with regard to ensuring consistent use of the most effective BCT categories nationwide. Specifically, the BCT categories reward and threat and identity were not included in all websites despite evidence that these categories contribute to higher cessation rates [13,23,24]. In addition, the list of ways in which the BCTs were implemented offers smoking cessation website programmers with the ability to test out and prioritize ideas about how they might want to incorporate and expand on the BCT categories for their individual program. In short, the BCTs ensure the quality of the web-based programs.

It is well-established in the smoking cessation literature that interactive features and tailored content lends to higher user engagement, which subsequently lends to higher cessation success outcomes [25-27]. In this regard, the use of BCTs alone are likely not enough. Instead, making the BCTs as engaging and as tailored as possible is another necessary step forward. For example, sharing health consequences in the form of a text-based list will not likely engage an end user as much as an interactive and graphical map that displays how different parts of the body are impacted by smoking.

### Limitations

There are a few limitations to this study that must be noted. First, the BCT taxonomy is vast and complex, requiring training to be well versed in understanding them. Although this research team consisted of a member who engaged in BCT training, interpreting which BCT applies to which web content may result in subjective nuances in representation. In addition, some website sections in Quebec were in the French language and required the use of Google Translate, possibly limiting the appropriateness of the content’s translation. Second, some website sections could not be accessed without creating an account and were therefore not included in the analysis, meaning that some BCT categories or BCTs were missed or not represented. Some websites also had accompanying resources (eg, account-based features); the use of BCTs in these resources was not assessed, which adds a layer of complexity. Third, the top BCTs found in Canadian websites may not be appropriate for international contexts due to differences in policies, laws, and tobacco use and cessation attitudes. Fourth, different BCTs may be more prevalent for different topics (eg, nutrition); therefore, BCT analyses should be specific to the topic at hand and should be analyzed and applied according to the needs of the behavior being investigated. Fifth, the success rates of each individual website (eg, based on user traffic) remains unknown; hence, it is difficult to make any assertions about how effective these websites are in relation to the BCTs without further evaluation. Sixth, the websites may have changed since the time of the analysis. Finally, websites were evaluated on just one occasion; for a more comprehensive assessment, each website/service would have to be used across multiple occasions (eg, as a quitter would use it).

### Conclusion

Analyzing the BCTs that underpin government-funded smoking cessation websites in Canada is an appropriate method for identifying strengths and weaknesses in these programs for influencing the target behavior of quitting smoking. The findings offer programmers and researchers with tangible directions for prioritizing and enhancing provincial and national smoking cessation programs, and an evaluation framework to assess smoking cessation outcomes in relation to the web-based content. The findings would benefit from being included in national conversations around how to implement and evaluate evidence-based smoking cessation support nationwide.

## Abbreviations

BCT: behavior change technique
